# Specificity in Legume-Rhizobia Symbioses

**DOI:** 10.3390/ijms18040705

**Published:** 2017-03-26

**Authors:** Mitchell Andrews, Morag E. Andrews

**Affiliations:** Faculty of Agriculture and Life Sciences, Lincoln University, PO Box 84, Lincoln 7647, New Zealand; moragandrews@gmail.com

**Keywords:** Leguminosae, N_2_ fixation, nodulation, *nod* genes, lateral gene transfer

## Abstract

Most species in the Leguminosae (legume family) can fix atmospheric nitrogen (N_2_) via symbiotic bacteria (rhizobia) in root nodules. Here, the literature on legume-rhizobia symbioses in field soils was reviewed and genotypically characterised rhizobia related to the taxonomy of the legumes from which they were isolated. The Leguminosae was divided into three sub-families, the Caesalpinioideae, Mimosoideae and Papilionoideae. *Bradyrhizobium* spp. were the exclusive rhizobial symbionts of species in the Caesalpinioideae, but data are limited. Generally, a range of rhizobia genera nodulated legume species across the two Mimosoideae tribes Ingeae and Mimoseae, but *Mimosa* spp. show specificity towards *Burkholderia* in central and southern Brazil, *Rhizobium*/*Ensifer* in central Mexico and *Cupriavidus* in southern Uruguay. These specific symbioses are likely to be at least in part related to the relative occurrence of the potential symbionts in soils of the different regions. Generally, Papilionoideae species were promiscuous in relation to rhizobial symbionts, but specificity for rhizobial genus appears to hold at the tribe level for the Fabeae (*Rhizobium*), the genus level for *Cytisus* (*Bradyrhizobium*), *Lupinus* (*Bradyrhizobium*) and the New Zealand native *Sophora* spp. (*Mesorhizobium*) and species level for *Cicer arietinum* (*Mesorhizobium*), *Listia bainesii* (*Methylobacterium*) and *Listia angolensis* (*Microvirga*). Specificity for rhizobial species/symbiovar appears to hold for *Galega officinalis* (*Neorhizobium galegeae* sv. *officinalis*)*, Galega orientalis* (*Neorhizobium galegeae* sv. *orientalis*), *Hedysarum coronarium* (*Rhizobium sullae*), *Medicago laciniata* (*Ensifer meliloti* sv. *medicaginis*), *Medicago rigiduloides* (*Ensifer meliloti* sv. *rigiduloides*) and *Trifolium ambiguum* (*Rhizobium leguminosarum* sv. *trifolii*). Lateral gene transfer of specific symbiosis genes within rhizobial genera is an important mechanism allowing legumes to form symbioses with rhizobia adapted to particular soils. Strain-specific legume rhizobia symbioses can develop in particular habitats.

## 1. Introduction

The Leguminosae (Fabaceae, the legume family) is comprised of ca. 19,300 species within 750 genera that occur as herbs, shrubs, vines or trees in mainly terrestrial habitats and are components of most of the world’s vegetation types [1,2,3]. Currently, the legume family is divided into three sub-families, the Caesalpinioideae, Mimosoideae and Papilionoideae [3,4]. Members of the Caesalpinioideae are grouped into four tribes, the Caesalpinieae, Cassieae, Cercideae and Detarieae comprising ca. 170 genera and 2250 species. The Mimosoideae are grouped into two tribes, the Ingeae and Mimoseae with ca. 80 genera and 3270 species, while the Papilionoideae consists of 28 tribes with ca. 480 genera and 13,800 species. However, a new classification of the legumes has been proposed with six sub-families based on the plastid *matK* gene sequences from ca. 20% of all legume species across ca. 90% of all currently recognized genera [5]. The six sub-families proposed are a re-circumscribed Caesalpinioideae, Cercidoideae, Detarioideae, Dialioideae, Duparquetioideae and Papilionoideae. In this system, the currently recognized Mimosoideae is a distinct clade nested within the re-circumscribed Caesalpinioideae. Species within the Cercidoideae, Detarioideae, Dialioideae and Duparquetioideae do not nodulate [5,6].

Most legume species can fix atmospheric nitrogen (N_2_) via symbiotic bacteria (general term “rhizobia”) in root nodules, and this can give them an advantage under low soil nitrogen (N) conditions if other factors are favourable for growth [7,8]. Furthermore, N_2_ fixation by legumes can be a major input of N into natural and agricultural ecosystems [9,10,11,12]. Generally, legume nodules can be classified as indeterminate or determinate in growth [13]. Indeterminate nodules maintain meristematic tissue, while determinate nodules have a transient meristem. Nodule type is dependent on host plant, and legume species that can produce both determinate and indeterminate nodules are rare [14,15]. All genera examined in the Caesalpinioideae and Mimosoideae had indeterminate nodules [13]. Within the Papilionoideae, most tribes had indeterminate nodules, but the Desmodieae, Phaseoleae, Psoraleae and some members of the Loteae show “desmodioid” determinate nodules and the Dalbergieae “aeschynomenoid” determinate nodules [13]. Desmodioid nodules have lenticels, and rhizobia “infected” tissue within them also contains uninfected cells. Aeschynomenoid nodules do not have lenticels, have uniform infected tissue and are always associated with lateral or adventitious roots. Where tested, species within the Desmodieae, Phaseoleae and Psoraleae had ureides as the main N-containing compound transported from nodules, but species in the Dalbergieae and Loteae transported amides/amino acids [13]. Indeterminate nodules have a single or branched apical meristem, and a few genera, such as *Lupinus* (Genisteae) and *Listia* (Crotalaria), have “lupinoid” nodules with two or more lateral meristems, which in some cases completely surround the subtending root [16,17]. Generally, indeterminate nodules have a mixture of infected and uninfected cells in the central nodule tissue, but lupinoid nodules, as for aeschynomenoid nodules (Dalbergiae), have uniformly-infected cells.

The nodulation process for almost all legumes studied is initiated by the legume production of a mix of compounds, mainly flavonoids, which induce the synthesis of NodD protein in rhizobia [18,19]. Different legumes produce different types/mixes of compounds. The NodD protein activates the transcription of other genes involved in the nodulation process, including those required to produce Nod factors, the signal molecules produced by the rhizobia and detected by the plant, which induce nodule organogenesis [20]. The *nodABC* genes encode for the proteins required to make the core Nod factor structure [18]. Nod factors from different rhizobia have a similar structure of a chitin-like N-acetyl glucosamine oligosaccharide backbone with a fatty acyl chain at the non-reducing end, but differ in their length of N-acetyl glucosamine oligosaccharide backbone and the length and saturation of the fatty acid chain. The Nod-factor core is modified by species-specific proteins, which results in various substitutions, including acetylation, glycosylation, methylation and sulfation. Perception of the Nod-factor signal in legumes is mediated by Nod factor receptors, which are plasma membrane localized serine/threonine receptor kinases in the case of the model legumes *Lotus japonicus* and *Medicago truncatula* [18,19].

The available data indicate that rhizobia enter the roots of most legume species via root hair infection [13]. Here, rhizobia enter root hairs, and host cell wall material grows around the developing “infection”, forming an infection thread, which grows through the cortex of the root, branching repeatedly. Generally, rhizobia are released from the tips of these infection threads into membrane-bound structures within host cells called symbiosomes where they differentiate into their N_2_-fixing form known as bacteroids. However, all species examined in the Caesalpinioideae, except herbaceous *Chamaecrista* spp. and a few species in the Papilionoideae, retain their rhizobia within infection threads [2,13]. Bacteroids vary greatly in their level of differentiation and viability depending on the legume host [13,21]. The process of root hair infection can lead to the formation of either indeterminate or desmodioid determinate nodules. A second mode of rhizobial infection occurs with species in the Dalbergiae (aeschynemonoid nodules) where rhizobia enter roots at the sites of lateral root emergence (“crack” entry), and infection threads are not involved in the infection process [22,23]. Thirdly, for at least some members of the Genisteae (e.g., *Lupinus* spp.) and Crotalariae (e.g., *Listia* spp.), rhizobia enter the roots directly through the root epidermis at the junction between epidermal cells, and again, infection threads are not involved in the infection process [17,21,24].

Over the past twenty-five years, DNA-based methods have become increasingly used to characterize rhizobia. In particular, phylogenetic analyses of sequences of the 16S ribosomal RNA (rRNA) gene, a range of “housekeeping” genes and genes involved in the symbiosis have been developed as a “standard approach” [15,25,26]. The 16S rRNA gene sequence on its own can delineate rhizobia at the genus level [27]. The main symbiosis genes studied are the “*nif*” genes, which encode the subunits of nitrogenase, the rhizobial enzyme that fixes N_2_, and the “*nod*” genes, which encode Nod factors that induce various symbiotic responses on legume roots. Specific *nod* genes have been shown to be major determinants of legume host specificity [28,29]. The *nif* and *nod* genes are often carried on plasmids or symbiotic islands, and these genes can be transferred (lateral transfer) between different bacterial species within a genus and more rarely across genera [30,31,32]. Almost all rhizobia tested had *nod* genes. However, a few *Bradyrhizobium* strains, which do not possess *nodABC* genes, can form N_2_ fixing nodules on particular *Aeschynomene* spp. [33,34]. Bacterial species from a range of genera in the α-proteobacteria (most commonly *Bradyrhizobium*, *Ensifer* (*Sinorhizobium*), *Mesorhizobium* and *Rhizobium*) and two genera in the β-proteobacteria (*Burkholderia* (*Paraburkholderia*) and *Cupriavidus*) can form functional (N_2_ fixing) nodules on specific legumes (Table 1, Table 2, Table 3 and Table 4). Reports that *Achromobacter* and *Herbaspirillum* (β-proteobacteria) produce N_2_ fixing nodules on *Prosopis juliflora* and *Aspalathus linearis*, respectively [35,36] and *Pseudomonas* (Gammaproteobacteria) produces N_2_ fixing nodules on *Robinia pseudoacacia* [37] and *Acacia confusa* [38] have not been confirmed. Furthermore, for *Lotus corniculatus*, *Geobacillus* (phylum Firmicutes), *Paenibacillus* (Firmicutes) and *Rhodococcus* (Actinobacteria) were for the first time reported as rhizobial symbionts [39]. These bacterial species had similar *nodA* gene sequences to *Mesorhizobium* isolated from the same plants, and it was concluded that the lateral gene transfer of these genes had occurred from the *Mesorhizobium*. However, lateral gene transfer of symbiosis genes is much less common between than within genera, and this work needs to be independently verified.

Legume species differ greatly in their specificity for rhizobial symbionts. *Galega officinalis* (tribe Galegeae) and *Hedysarum coronarium* (tribe Hedysareae) have been highlighted as being highly specific with respect to their rhizobial symbionts [120,145,296,297]. Both of these species are in the inverted repeat lacking clade (IRLC). The IRLC is marked by the loss of one copy of the inverted region of the plastid genome [298,299]. Almost all genera in the IRLC are temperate; all have indeterminate nodules, and where examined, their bacteroids were terminally differentiated and could not return to their bacterial form [13]. The IRLC contains several important temperate grain (e.g., *Pisum sativum* and *Vicia faba*) and forage (e.g., *Trifolium* spp. and *Medicago* spp.) legumes. There is evidence that at least some of these crop legumes have a high degree of rhizobial specificity. For example, an analysis of core and symbiotic genes of rhizobia nodulating *Vicia faba* and *Vicia sativa* from different continents showed that they belong to a phylogenetically-compact group indicating that these species are restrictive hosts [117]. In contrast, *Macroptilium purpureum* and the grain legumes *Phaseolus vulgaris* and *Vigna unguiculata* in the tribe Phaseoleae are nodulated by rhizobia from different genera across the α- and β-proteobacteria [264,283,300]. The Phaseoleae are of tropical/subtropical origin, have desmodioid determinate nodules with bacteroids, which are not terminally differentiated [2,13].

Here, the literature on legume-rhizobia symbioses in field soils was reviewed and genotypically characterised rhizobia related to the taxonomy of the legumes from which they were isolated. The objectives of the work were to collate data on legume rhizobia symbioses and then assess to what extent legume specificity for rhizobial symbionts is related to legume taxonomy.

## 2. Framework and Assumptions of Study

The general classification of the Leguminosae follows Lewis et al., 2005 [1], with updates [3,4]. The sub-families Caesalpinioideae, Mimosoideae and Papilionoideae are considered separately. The Papilionoideae is split into those that show indeterminate nodules and those that show determinate nodules. Those that show indeterminate nodules are further split into the IRLC and all other clades.

Nodulating bacteria were classified at the genus level, on the basis of sequences of the 16S rRNA gene (almost all cases), and/or the 16S–23S DNA intergenic spacer region, and/or common house-keeping genes, and/or DNA-DNA hybridisations, and these results are presented in the tables. Sequences for *nif* and *nod* genes are considered in the text. Rhizobial genus and species names validated in the International Journal of Systematic and Evolutionary Microbiology were used with one exception: *Burkholderia* was retained as opposed to using *Paraburkholderia* [301,302], as a case to reinstate *Burkholderia* is being prepared by workers in the field. The term symbiovar (sv.) is used when describing rhizobial strains within the same species that differ with respect to the legume species they effectively nodulate [303].

A comprehensive collation of published legume-rhizobia symbioses up until 30 September 2016 was carried out. Articles were collected by searching the Institute for Scientic Information (ISI) Web of Science using each legume genus partnered with each of the rhizobia, *Bradyrhizobium*, *Burkholderia*, *Cupriavidus*, *Ensifer*, *Mesorhizobium*, *Rhizobium* and *Sinorhizobium* as the keywords. Further searches were carried out on the literature quoted in the selected papers and those listed as quoting the selected papers in the ISI Web of Science. Only data for plants sampled under field conditions, or for plants grown in soils taken from the field, or supplied field soil extracts were used. Bacteria isolated from legume nodules were accepted as rhizobia if they were shown to produce functional (N_2_ fixing) nodules on inoculation of their original legume host or a species within the original host legume genus under axenic conditions. The range of measurements and visual assessments used as evidence of the occurrence of N_2_ fixation were accepted. These were acetylene reduction activity, red/pink nodules (evidence of leghaemoglobin and, hence, nodules assumed to be active), increased total plant or shoot dry matter or N content, visually greener (increased chlorophyll) and increased plant vigour. However, it is acknowledged that in some cases, greater growth, vigour and/or greenness could have been caused by plant hormone production by the bacterium [304]. All data obtained for all species are presented with three exceptions. Representative data are presented for *Glycine max*, *Phaseolus vulgaris* and *Vigna unguiculata* due to the large number of publications on these three species.

## 3. Caesalpinioideae-Rhizobia Symbioses

Of the three legume sub-families, the Caesalpinioideae contains the smallest proportion of nodulated genera with nodulation confirmed for *Campsiandra*, *Chidlowia*, *Dimorphandra*, *Erythrophleum*, *Jacqueshuberia*, *Melanoxylon*, *Moldenhauwera* and *Tachigali* in the tribe Caesalpinieae and *Chamaecrista* in the tribe Cassieae [2,13]. Only two studies have genotypically characterised bacteria confirmed as rhizobia of Caesalpinioideae species. Firstly, five rhizobial isolates from *Dimorphandra wilsonii* and one from *Dimorphandra jorgei* sampled in the Cerrado biome in Brazil were *Bradyrhizobium* [305]. Secondly, 166 rhizobial isolates from *Erythrophleum fordii* sampled at four sites in the Guangdong and Guangsii Provinces of the southern sub-tropical region of China were also all *Bradyrhizobium* [306]. In both studies, core and symbiosis gene sequences indicated that there were diverse and novel strains amongst the isolates.

Data are available for bacterial isolates from nodules of other Caesalpinioideae species, but their ability to produce N_2_ fixing nodules on their legume host under axenic conditions was not tested. Specifically, three isolates from *Tachigali versicolor* sampled on Barro Colorado Island, Panama, which were not tested on their original host plant, but were shown to nodulate *Macroptilium atropurpureum*, were *Bradyrhizobium* [307]. Similarly, strain STM934, stated to be confirmed as *Bradyrhizobium*, was isolated from nodules of *Erythrophleum guineensis* growing in natural forests of the Ziama reservation in southeast Guinea and shown to produce functional nodules on *Macroptilium atropurpureum* [308]. In this case, a re-inoculation experiment was carried out on the original host, but the substrate was non-sterile forest soil. *Bradyrhizobium* was isolated from and shown to nodulate *Chamaecrista* sampled in Kakadu National Park, Northern Territory, Australia, but N_2_ fixation was not reported [309]. Furthermore, there are several reports that *Bradyrhizobium* inoculum can increase nodulation of *Chamaecrista* spp. under field conditions in Australia and China [310,311,312]. Thus, the available evidence indicates that *Bradyrhizobium* spp. are the dominant, possibly exclusive, rhizobial symbionts of legumes in the Caesalpinioideae, but data are limited, and the degree of specificity between legumes in the Caesalpinioideae and their rhizobial symbionts cannot be assessed without further work.

## 4. Mimosoideae-Rhizobia Symbioses

Rhizobia have been characterized from 15 species across seven genera in the tribe Ingeae and ca. 120 species from 13 genera in the tribe Mimoseae within the sub-family Mimosoideae (Table 1). *Bradyrhizobium*, *Ensifer*, *Mesorhizobium* and *Rhizobium* were each reported to nodulate species in the Ingeae and the Mimoseae. Furthermore, *Ochrobactrum* was reported to nodulate *Acacia* *mangium* (Ingeae); *Allorhizobium* and *Devosia* were reported to nodulate *Neptunia natans* (Mimoseae); and there are many reports that *Cupriavidus* and *Burkholderia* nodulate *Mimosa* spp. and related species (Mimoseae) (Table 1). In addition, excepting *Acacia auriculiformis* (Ingeae) and *Mimosa pigra* (Mimoseae), all species that were examined in three or more separate studies, *Acacia mangium*, *Acacia saligna*, *Calliandra grandiflora* and *Senegalia senegal* (Ingeae), *Leucaena leucocephala*, *Mimosa diplotricha*, *Mimosa pudica*, *Parapiptadenia rigida*, *Prosopis alba* and *Vachellia* *tortilis* (Mimoseae), were nodulated by at least three different rhizobial genera. Thus, a range of rhizobial genera, including both α- and β-proteobacteria, can nodulate legume species across the two Mimosoideae tribes, and generally, where tested over different studies, species within the Ingeae and Mimoseae tribes were promiscuous with respect to their rhizobial symbionts.

The *Mimosa* species examined across studies were the pan-tropical invasive *Mimosa diplotricha* and *Mimosa pudica*, and findings for these species appear not to reflect the situation with most *Mimosa* spp., which are endemic with a restricted range. The evidence indicates that most *Mimosa* spp. show specificity towards the rhizobial genus depending on their distribution with *Burkholderia*, *Rhizobium*/*Ensifer* and *Cupriavidus*, the main rhizobial symbiont of endemic *Mimosa* spp. in central and southern Brazil, central Mexico and southern Uruguay, respectively [73,74,79]. The 16S rRNA and housekeeping gene sequences were diverse for *Mimosa* *Burkholderia* symbionts in Brazil, *Rhizobium*/*Ensifer* in Mexico and *Cupriavidus* in Uruguay. For *Burkholderia* in Brazil and *Rhizobium/Ensifer* in Mexico, the symbiosis gene sequences were largely congruent with the 16S rRNA and housekeeping gene sequences, indicating that these genes diverged over a long period within *Burkholderia* without substantial horizontal gene transfer between species [73,74]. For *Cupriavidus* rhizobia in Uruguay, the *nodA* gene sequences were not congruent with the housekeeping gene sequences, but grouped together in a cluster [79]. This is strong evidence that the various *Cupriavidus* species obtained their symbiosis genes via within group lateral gene transfer [32,79,313]. It is not known if endemic *Mimosa* spp. nodulated by a particular rhizobia genus can form N_2_-fixing symbioses with *Mimosa* rhizobia of different genera from outside their region.

## 5. Papilionoideae-Rhizobia Symbioses

### 5.1. The IRLC

Data are available for 103 species from 27 genera/five tribes in the IRLC with *Ensifer*, *Mesorhizobium* and *Rhizobium*, commonly, and *Bradyrhizobium*, *Neorhizobium* and *Phyllobacterium*, rarely, reported to nodulate species within this clade (Table 2). There are no reports of *Burkholderia* or *Cupriavidus* symbionts within the IRLC. Previously, *Galega officinalis*, *Galega orientalis* and *Hedysarum coronarium* within the IRLC clade were reported to only form effective nodules with their respective symbionts *Neorhizobium galegeae* sv. *officinalis*, *Neorhizobium galegeae* sv. *orientalis* and *Rhizobium sullae* [120,145,296,297]. The data in Table 2 indicate two other specific relationships between IRLC legumes and rhizobia. Firstly, eight separate studies on *Cicer arietinum* carried out over different countries and continents reported *Mesorhizobium* as the only symbiont. The 16S rRNA and housekeeping gene sequences indicated that strains of *Mesorhizobium ciceri* and *M*. *mediterraneum* were common, but not exclusive *Mesorhizobium* symbionts of *Cicer arietinum* in most studies outside China, with *M*. *muleiense* the main symbiont in Northwest China [100]. Across studies where tested, *nifH* and *nodC* gene sequences were similar for all *Mesorhizobium* isolates shown to produce functional nodules on *Cicer arietinum*, indicating their specificity towards this legume species and that lateral transfer of these genes had occurred between the different *Mesorhizobium* spp. [97,99,100].

Secondly, for the tribe Fabeae, seventeen studies across five *Lathyrus* species, *Lens culinaris*, *Pisum sativum* and eleven *Vicia* spp. reported *Rhizobium* as the only symbiont. Across these studies, *Rhizobium leguminosarum* (and where tested, *R. leguminosarum* sv. *viciae*) was the most common symbiont with some varieties of *Pisum sativum*, such as cv. Afghanistan, only nodulated by specific strains of *Rhizobium leguminosarum* sv. *viciae*, which occur in soils in their native range in Afghanistan/Turkey [103]. Furthermore, in a study of 154 isolates of 18 *Vicia* species grown in 16 Chinese provinces, only 17 representative *Rhizobium Leguminosarum* sv. *viciae* isolates, from a wide range of potential rhizobia, produced fully-developed, effective (“colour red”) nodules [115]. Thus, a highly specific relationship has developed between species in the Fabeae and *R. leguminosarum* sv. *viciae*, but it is not an exclusive relationship, as *R. fabeae* [116], *R. multihospitium* [105], *R. pisi* [111], *R. laguerreae* [314] and *R. anhuiense* [108] have been reported to effectively nodulate Fabeae species. However, the *nifH* and *nodC* gene sequences of all of these rhizobia showed high similarity, indicating their specificity towards the Fabeae species and that, in this case, lateral gene transfer had occurred between different *Rhizobium* spp. [32,108].

Within the tribe Trifolieae, 14 out of 16 *Medicago*/*Melilotus* spp. had *Ensifer* as symbiont, but in four cases not exclusively. The most studied species, *Medicago sativa* (lucerne), was commonly nodulated by *Ensifer meliloti*, which is the recommended inoculum for this legume crop, but it also had *Neorhizobium* and *Rhizobium* symbionts. However, *Medicago laciniata* and *Medicago rigiduloides* were found to only nodulate with *Ensifer* strains sampled in their native range in the Mediterranean Basin. These strains were formally described as *Ensifer meliloti* sv. *medicagini*s and *Ensifer meliloti* sv. *rigiduloides*, respectively [148,153]. Similarly, different *Trifolium* spp. have different compatibility with different strains of *Rhizobium leguminosarum* sv *trifolii*, which is the recommended inoculum for most *Trifolium* crops. *Trifolium ambiguum*, in particular, has been highlighted as only forming N_2_-fixing nodules with strains of *Rhizobium leguminosarum* sv *trifolii* specific to its region of origin (the Caucasus and Eastern Europe) [315].

In relation to other members of the IRLC, *Ensifer*, *Mesorhizobium* and *Rhizobium* were shown to nodulate species within *Astragalus*, *Colutea*, *Glycyrrhiza*, *Oxytropis* and *Sphaerophyceae* (Galegeae), *Hedysarum* (Hedysareae) and *Trifolium*. Furthermore, *Astragalus adsurgense*, *Astragalus complanatus*, *Colutea arborescens*, *Oxytropis glabra* and *Sphaerophysa salsula* (Galegeae), *Caragana intermedia* (Hedysareae) and *Trifolium fragiferum* and *Trifolium repens* were all nodulated by three different rhizobial genera. Thus, for the IRLC, specificity for the rhizobial genus appears to hold at the tribe level for the Fabeae (*Rhizobium* spp.) and species level for *Cicer arietinum* (*Mesorhizobium* spp.). Specificity for rhizobial species or symbiovar holds for *Galega officinalis* (*Neorhizobium galegeae* sv. *officinalis*), *Galega orientalis* (*Neorhizobium galegeae* sv. *orientalis*), *Hedysarum coronarium* (*Rhizobium sullae*), *Medicago laciniata* (*Ensifer meliloti* sv. *medicaginis*), *Medicago rigiduloides* (*Ensifer meliloti* sv. *rigiduloides*) and *Trifolium ambiguum* (*Rhizobium leguminosarum* sv. *trifolii*), but it is not a characteristic of all members of the clade.

### 5.2. Clades with Indeterminate Nodules, Excluding the IRLC

Data are shown for 113 species from 33 genera across 13 Papilionoideae tribes with indeterminate nodules that do not show the IRLC mutation (Table 3). *Azorhizobium*, *Bradyrhizobium*, *Burkholderia*, *Ensifer*, *Mesorhizobium*, *Methylobacterium*, *Microvirga*, *Neorhizobium*, *Ochrobactrum*, *Pararhizobium*, *Phyllobacterium* and *Rhizobium* were all reported to nodulate species within this group. *Amorpha fruticosa* and *Dalea purpurea* (Amorpheae), *Retama sphaerocarpa* and *Spartium junceum* (Genisteae), *Coronilla varia* (Loteae), *Tephrosia falciformis* and *Tephrosia villosa* (Millettiea), *Gliricidia sepium* and *Robinia pseudoacacia* (Robineae) and *Sesbania sericea* and *Sesbania virgata* (Sesbanieae) were nodulated by two rhizobial genera. *Aspalathus linearis* and *Crotalaria pallida* (Crotalarieae), *Tephrosia purpurea* (Millettiea), *Sesbania cannabina*, *Sesbania punicea*, *Sesbania rostrata* and *Sesbania sesban* (Sesbanieae), *Sophora alopecuroides* and *Sophora flavescens* (Sophoreae) and *Ammopiptanthus nanus* and *Ammopiptanthus mongolicus* (Thermopsideae) were all nodulated by at least three different rhizobial genera. Thus, generally, where tested, Papilionoideae species with indeterminate nodules excluding the IRLC were promiscuous in relation to rhizobial symbiont. Within the Genisteae, *Bradyrhizobium* was the only symbiont reported for nine *Cytisus* spp. across ten separate studies and three *Genista* spp. across three separate studies (Table 3). Furthermore, eight out of 10 *Lupinus* spp. (Genisteae) across 11 separate studies were nodulated by *Bradyrhizobium*. These results indicate that *Bradyrhizobium* may be the main symbionts of Genisteae species, but further work is required to confirm this. Generally, 16S rRNA and housekeeping gene sequences indicate that diverse *Bradyrhizobium* spp. form N_2_-fixing nodules on *Cytisus* spp. and *Lupinus* spp., but the diversity of their symbiosis genes is dependent on the geographical origin of the legumes. For example, most *Bradyrhizobium* isolates from native *Lupinus* spp. in Europe form a distinct lineage, ‘clade 11’, on the basis of their *nodA* gene sequences [180,195]. Similarly, different *Bradyrhizobium* spp. associated with native *Cytisus villosus* in Morocco all showed similar *nodC* and *nifH* sequences, which were closely related to those of *Bradyrhizobium japonicum* sv. *genistearum* [175]. In contrast, rhizobia sampled from invasive *Cytisus scoparius* sampled in six states in the United States, differed with respect to housekeeping and symbiosis gene sequences [174]. Specifically, one group of isolates had both housekeeping and symbiosis gene sequences similar to a *Bradyrhizobium* clade from native legumes in Western North America, but two clades had *nifD*, *nifH* and *nodC* sequences highly similar or identical to a *Cytisus scoparius* strain isolated in Spain, while their housekeeping genes were similar to American *Bradyrhizobium* clades. Thus, it appears that *Bradyrhizobium* ancestrally associated with native North American legumes have acquired symbiosis genes from European *Cytisus scoparius* *Bradyrhizobium* symbionts via lateral gene transfer.

The two exceptions to *Bradyrhizobium* as the rhizobial symbiont of *Lupinus* spp. were *Ochrobactrum* [181] and *Microvirga* [166], both of which are rare as rhizobial symbionts of legumes. The *nodD* sequence for *Ochrobactrum* and the *nodA* sequence for *Microvirga* indicated that both bacteria obtained their *nod* genes via horizontal gene transfer from more common rhizobial genera. *Microvirga* is also the only bacterial genus shown to form N_2_-fixing nodules on *Listia angolensis* [17]. Similarly, *Listia bainesii* has been found to only produce N_2_-fixing nodules with pink pigmented *Methylobacterium* [17]. It was suggested that the seasonally waterlogged habitat of *Listia* spp. may have resulted in the selection of unusual rhizobial symbionts adapted to their environments [17].

In one study in the Cape Floristic Region (CFR) of South Africa, *Burkholderia* was reported to be the exclusive symbiont of ten *Cyclopia* spp., *Podalyria* *calyptera* and *Virgilia oroboides*, all species in the Podalyrieae plus three *Hypocalyptus* spp. (Hypocalypteae) [191]. *Burkholderia* was confirmed to nodulate *Podolyria calyptrata* and *Virgilia oroboides* in the CFR [136,193,197]. The majority of *Burkholderia* isolates had unique *nifH* and *nodA* gene sequences, and the specificity of these symbioses needs testing.

Previously, *Sesbania sesban* was reported to be highly promiscuous with respect to rhizobial symbionts [31], and the data here indicate that this could be a genus level trait (Table 3). However, the reports that *Sophora alopecuroides* and *Sophora flavescens* sampled in China are nodulated by *Ensifer*, *Mesorhizobium*, *Phyllobacterium* and *Rhizobium* with a wide range of symbiosis gene sequences [207,208] contrasts with the finding that New Zealand (NZ) native *Sophora* spp. were exclusively nodulated by *Mesorhizobium* spp. with almost identical unique *nodA* and *nodC* gene sequences [210,316,317]. This emphasises that species within the same genus can vary greatly with respect to their specificity for rhizobial symbionts.

### 5.3. Clades with Determinate Nodules

The Dalbergieae are almost exclusively of tropical/sub-tropical distribution and show an aeschynomenoid determinate nodule structure [2]. Rhizobia have been characterised for 23 species from seven genera in the Dalbergieae, *Adesmia*, *Aeschynomene*, *Arachis*, *Centrolobium*, *Dalbergia*, *Pterocarpus* and *Zornia* (Table 4). *Bradyrhizobium* was found to nodulate all species, except *Adesmia bicolor* (*Rhizobium*), with *Rhizobium* also reported for *Arachis hypogaea* in two studies. Thus, on the data available, the Dalbergieae appear to be primarily nodulated by *Bradyrhizobium*. For *Arachis hypogaea*, twelve separate studies reported *Bradyrhizobium* as a rhizobial symbiont (Table 4). Across these studies, both core and symbiosis gene sequences indicated that *Arachis hypogaea* was nodulated by a diverse range of *Bradyrhizobium* spp. and are promiscuous with respect to *Bradyrhizobium* spp. Excepting *Arachis hypogaea*, data are limited for rhizobial symbionts of species in the Dalbergieae. However, the unusual ability of specific *Bradyrhizobium* strains that lack canonical *nodABC* genes to form N_2_-fixing nodules on roots and/or stems of particular *Aeschynomene* spp. is highlighted [34,215].

The closely related tribes Desmodieae, Phaseoleae and Psoraleae are also mainly of tropical/sub-tropical distribution, and with rare exceptions, species within these tribes showed a desmodioid nodule structure [2,15]. Rhizobia have been characterized for 25 species from three genera, *Desmodium*, *Kummerowia* and *Lespedeza*, in the Desmodieae (Table 4). Species from all three genera, *Desmodium microphyllum*, *Desmodium racemosum*, *Desmodium sequax*, *Kummerowia striata*, *Lespedeza bicolor* and *Lespedeza daurica*, were nodulated by rhizobia from three separate genera. Similarly, for 28 species across 14 genera within the Phaseoleae, there was no strong evidence for high specificity for rhizobial symbiont (Table 4). *Phaseolus vulgaris* and *Vigna unguiculata* have been highlighted as being promiscuous with respect to their rhizobial symbionts under field conditions. Data in Table 4 show that both species can be nodulated by different rhizobial genera in the α-proteobacteria, as well as *Burkholderia* in the β-proteobacteria. Across three studies, *Phaseolus lunatus* was reported to be nodulated by *Bradyrhizobium* and *Rhizobium*, while *Vigna angularis*, *Vigna radiata* and *Vigna subterranea* were reported to be nodulated by three separate rhizobial genera. Data are limited for other genera/species within the Phaseoleae with the exception of *Glycine max*, which is the main grain/oil seed legume grown worldwide, and *Glycine soja*. Both *Glycine* spp. were nodulated by *Bradyrhizobium*, *Ensifer* and *Rhizobium*. In the one case where separate studies were carried out on one species within the Psoraleae, *Psoralea pinnata* was nodulated by *Bradyrhizobium*, *Burkholderia* and *Mesorhizobium* [136,193,287]. Thus, where tested, species within the Desmodieae, Phaseoleae and Psoraleae were promiscuous with respect to their rhizobial symbionts.

Species in the Loteae, which show a desmodoid nodule structure, are of temperate distribution [2]. Data are available for 16 *Lotus* spp. within the Loteae across 13 separate studies. For all species examined in two or more studies, at least two rhizobia genera were reported as symbionts. Overall, the available data indicate that legume species with a desmodioid determinate nodule structure are promiscuous with respect to their rhizobia symbionts.

## 6. Legume Specificity for Rhizobial Symbionts

The objectives of the work were to collate data on legume rhizobia symbioses and assess the extent that legume specificity for rhizobial symbiont is related to legume taxonomy. *Bradyrhizobium* spp. were the exclusive rhizobial symbionts of species in the Caesalpinioideae; but, rhizobia were characterised for only three legume species over two studies, and the degree of specificity between legumes in the Caesalpinioideae and their rhizobial symbionts cannot be assessed without further work. Generally, species within the two Mimosoideae tribes, Ingeae and Mimoseae were promiscuous with respect to their rhizobial symbionts, but *Mimosa* spp. show specificity towards the rhizobia genus depending on their distribution, with *Burkholderia*, *Rhizobium*/*Ensifer* and *Cupriavidus* the main rhizobial symbiont of endemic *Mimosa* spp. in central and southern Brazil, central Mexico and southern Uruguay, respectively [73,74,79]. Papilionoideae species with indeterminate nodules were split into the IRLC and all other clades. A range of species within both groups nodulated with different rhizobia genera, but there was also strong evidence that some species within both groups showed specificity for rhizobial genus or species/symbiovar. Specificity for rhizobial genus appears to hold at the tribe level for the Fabeae (*Rhizobium*), the genus level for *Cytisus* (*Bradyrhizobium*), *Lupinus* (*Bradyrhizobium*) and NZ native *Sophora* spp. (*Mesorhizobium*) and the species level for *Cicer arietinum* (*Mesorhizobium*), *Listia bainesii* (*Methylobacterium*) and *Listia angolensis* (*Microvirga*). Specificity for rhizobial species/symbiovar appears to hold for *Galega officinalis* (*Neorhizobium galegeae* sv. *officinalis*), *Galega orientalis* (*Neorhizobium galegeae* sv. *orientalis*), *Hedysarum coronarium* (*Rhizobium sullae*), *Medicago laciniata* (*Ensifer meliloti* sv. *medicagin*is), *Medicago rigiduloides* (*Ensifer meliloti* sv. *rigiduloide*s) and *Trifolium ambiguum* (*Rhizobium leguminosarum* sv. *trifolii*). For Papilionoideae with determinate nodules, the Dalbergieae (aeschynomenoid nodules) were primarily nodulated by *Bradyrhizobium*, while those in the Desmodieae, Phaseoleae, Psoraleae and Loteae (desmodioid nodules) were promiscuous with respect to rhizobial genus. Thus, on the data available, species in the Papilionoideae that show specificity for rhizobial genus, species or symbiovar have indeterminate nodules and are generally (but not exclusively) of temperate distribution. However, many temperate legumes with indeterminate nodules are promiscuous with respect to the rhizobial genus indicating that high specificity for rhizobial symbiont only occurs under specific conditions [318].

For *Mimosa* spp., specificity towards rhizobial genus depending on distribution is likely to be at least in part related to the relative occurrence of the potential symbionts in soils of the different regions [74,79]. For example, evidence indicates that *Mimosa* *Cupriavidus* symbionts are absent from soils in central and southern Brazil, while *Mimosa* *Burkholderia* symbionts are absent from soils in southern Uruguay. This has been related to soil characteristics with low pH favouring *Burkholderia* over *Cupriavidus* in Brazil, but high heavy metal content favouring *Cupriavidus* over *Burkholderia* in Uruguay [73,74,79]. It was proposed that native *Mimosa* in the different regions have selected symbiotic bacteria adapted to local conditions, which resulted in the development of highly specific associations [74,79]. However, for this to occur, such rhizobia must be available in the soil. The within genus 16S rRNA and housekeeping gene sequences were diverse for *Mimosa* symbionts in all regions. For *Burkholderia* in Brazil and *Rhizobium/Ensifer* in Mexico, the symbiosis gene sequences were largely congruent with the 16S rRNA and housekeeping gene sequences [73,74]. For *Cupriavidus* rhizobia in Uruguay, the *nodA* gene sequences were not congruent with the housekeeping gene sequences, but grouped together in a cluster, indicating that the various species within the group obtained their symbiosis genes via within group lateral gene transfer [79]. Lateral gene transfer is a mechanism whereby rhizobia and non-rhizobial bacteria adapted to local soil conditions could become specific rhizobial symbionts of legumes growing in these soils. The evidence described above indicates that lateral gene transfer of symbiosis genes has been important within the Papilionoideae in relation to the development of specific relationships between the Fabeae and *Rhizobium* spp., *Cytisus* and *Bradyrhizobium* spp., *Lupinus* and *Bradyrhizobium* spp., NZ native *Sophora* and *Mesorhizobium* spp. and *Cicer arietinum* and *Mesorhizobium* spp. Data are presented for *Mesorhizobium* isolates from *Cicer arietinum* and NZ native *Sophora* spp. to emphasise this point.

**Figure 1:**
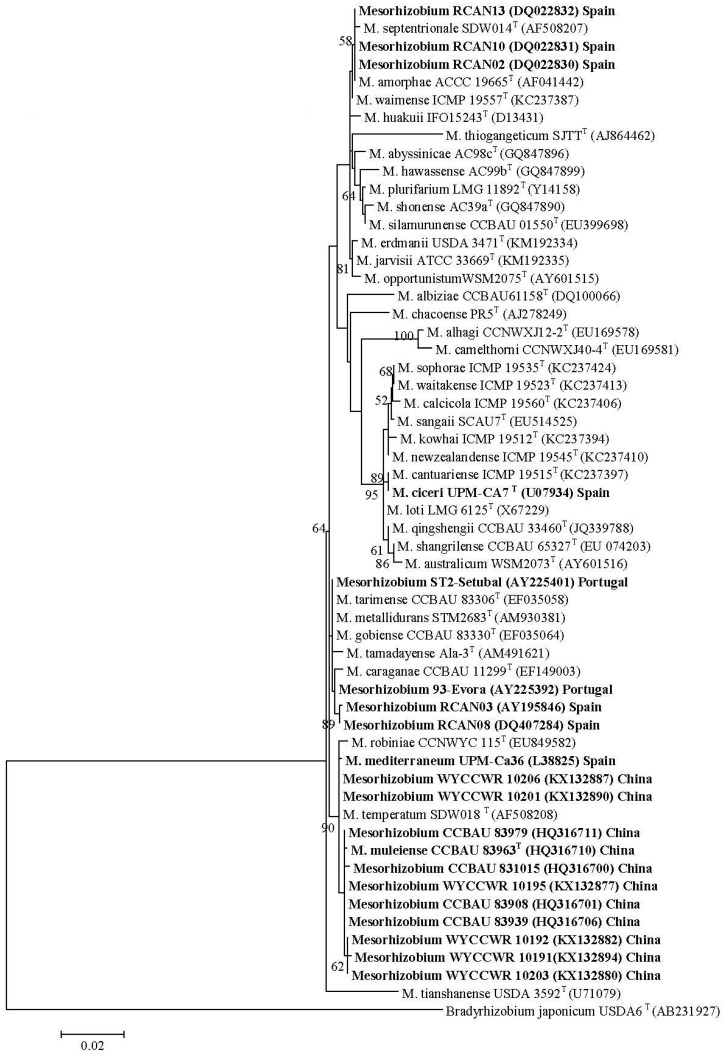

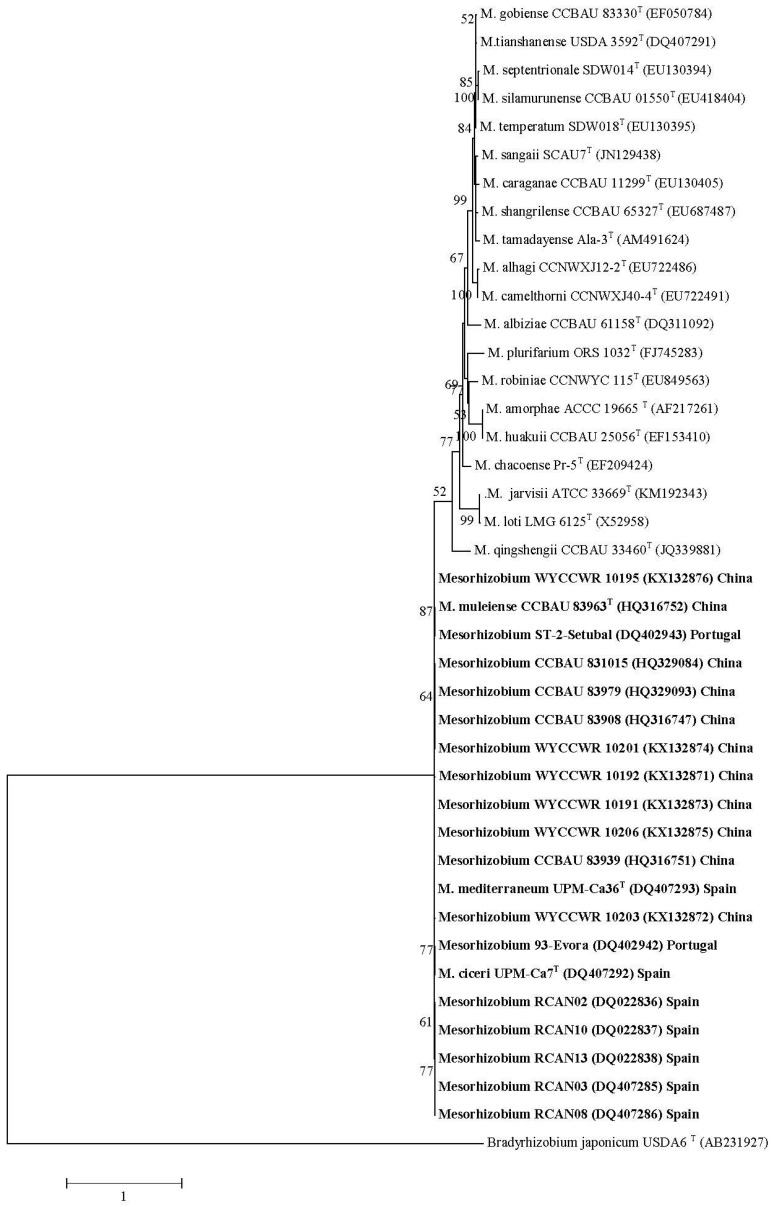
16S rRNA gene maximum likelihood (ML) tree (ca. 1360 bp) (**A**) and *nodC* gene ML tree (ca. 630 bp) (**B**) of rhizobial strains isolated from *Cicer arietinum* in Spain, Portugal and China (bold) and selected *Mesorhizobium* type strains [97,99,100]. Numbers on branches are bootstrap % from 1000 replicates (shown only when ≥50%).

**Figure 2:**
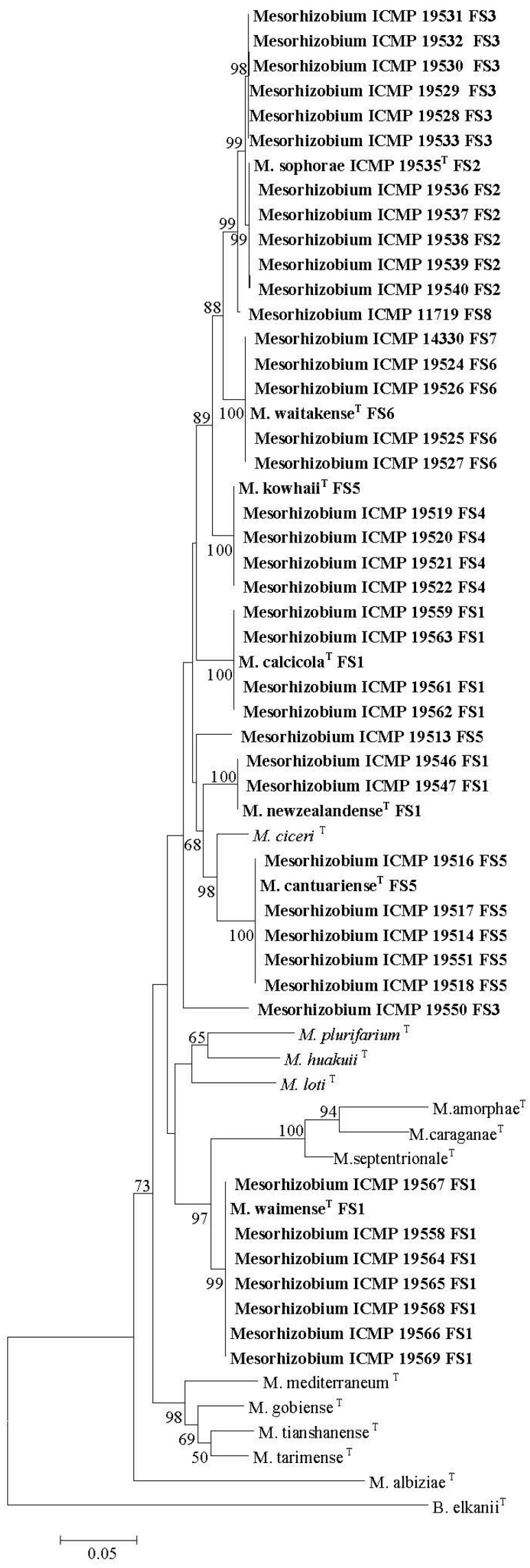

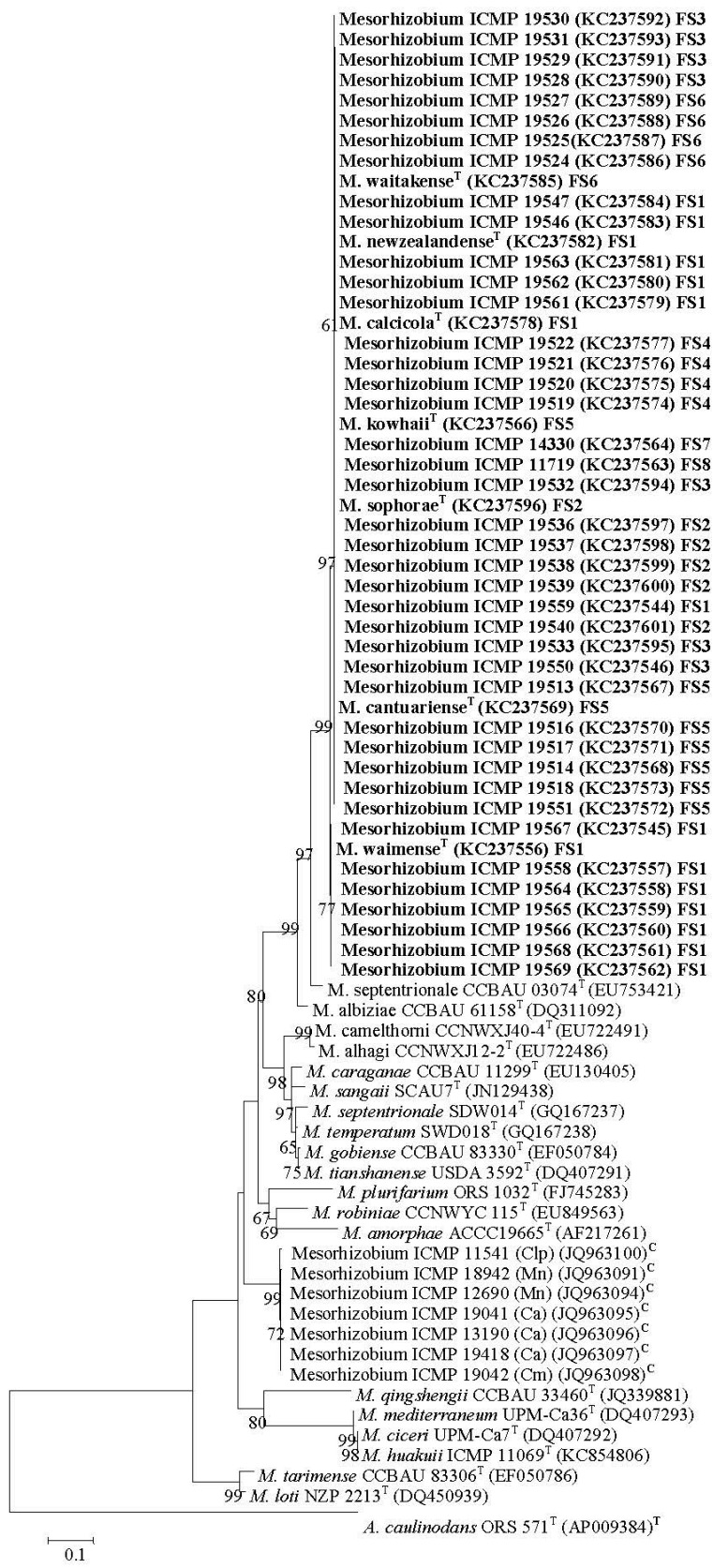
Concatenated *recA*, *gln11* and *rpoβ* gene maximum likelihood (ML) tree (ca. 1800 bp) (**A**) and *nodC* gene ML tree (ca. 650 bp) (**B**) of rhizobial strains isolated from New Zealand (NZ) native *Sophora* spp. (bold) and selected *Mesorhizobium* type strains. The *nodC* sequences of isolates from NZ native legumes *Clianthus puniceus* (Clp), *Montigena novozealandiae* (Mn), *Carmichaelia australis* (Ca) and *Carmichaelia monroi* (Cm) tested on NZ *Sophora* spp. are shown. Numbers on branches are bootstrap % from 500 replicates (shown only when ≥50%). FS = field site. Modified from Tan et al., 2015 [210].

Firstly, 20 *Mesorhizobium* isolates from *Cicer arietinum*, sampled across three countries in five separate studies, showed diverse 16S rRNA sequences, but highly similar *nodC* sequences (Figure 1A,B). Here, evidence is strong that native *Mesorhizobium muleiense* adapted to alkaline soils in Gansu and Xinjiang Provinces of China obtained its *Cicer arietinum*-specific symbiotic genes from *Mesorhizobium ciceri* or *Mesorhizobium mediterraneum* introduced together with *Cicer arietinum* used as a crop [100]. Secondly, 48 isolates from four NZ native *Sophora* spp. sampled at eight different field sites separated into eight groups and three individual isolates on the basis of their concatenated *recA*, *gln11* and *rpoβ* gene sequences, but showed almost identical *nodC* (and *nodA* [210]) sequences (Figure 2A,B). Seven of the groups have been formally identified as new species [316,317]. This relationship between NZ native *Sophora* spp. and *Mesorhizobium* spp. with specific symbiosis gene sequences is highly specific as none of twenty rhizobial isolates from common weed and crop legumes in NZ produced functional nodules on the NZ native *Sophora microphylla* [190]. Furthermore, *Mesorhizobium* isolates from *Carmichaelia*, *Clianthus* and *Montigena*, the only other NZ native legume genera, did not nodulate NZ native *Sophora microphylla* or *Sophora tetraptera* [127]*. Mesorhizobium* isolates from *Carmichael*ia, *Clianthus* and *Montigena* had unique *nodC* sequences different from those of *Sophora* *Mesorhizobium* isolates (Figure 2B). However, in some cases, their 16S rRNA, recA and *gln11* sequences were similar to those of *Sophora* *Mesorhizobium* isolates, emphasizing the importance of the specific symbiosis genes in the NZ *Sophora* *Mesorhizobium* symbiosis [137]. Generally, *Sophora* isolates from the same field site grouped together on concatenated *recA*, *gln11* and *rpoβ* gene sequences (Figure 2B). This apparent link between housekeeping gene sequences and field site is compatible with the proposal that lateral transfer of symbiosis genes to *Mesorhizobium* strains adapted to local soil conditions has occurred.

Some varieties of *Pisum sativum*, such as cv. Afghanistan, are only nodulated by specific strains of *Rhizobium leguminosarum* sv. *viciae*, which occur in soils in their native range in Afghanistan/Turkey [103]. The ability of these strains to nodulate *Pisum sativum* cv. Afghanistan is controlled by a single recessive gene, *sym2* in the plant. The *sym2* allele interacts with a specific gene, *nodX*, present in *R. leguminosarum* sv. *viciae* strains able to nodulate cv. Afghanistan [319]. The *nodX* gene product acetylates a Nod factor, which mediates a specific compatible interaction with this cultivar [320,321]. Furthermore, *Medicago laciniata and Medicago rigiduloides* were found to only nodulate with *Ensifer* *meliloti* strains (*E. meliloti* sv. *medicagini*s and *E. meliloti* sv. *rigiduloides*, respectively) sampled in their native range in the Mediterranean Basin [148,153], and *Trifolium ambiguum* only forms N_2_-fixing nodules with strains of *Rhizobium leguminosarum* sv. *trifolii* specific to its region of origin, the Caucasus and Eastern Europe [315]. The mechanisms of these highly specific relationships are not fully understood, but the *nodA* gene sequences of *E. meliloti* sv. *medicagini*s and *nodA*, *nodB* and *nodC* gene sequences of *E. meliloti* sv. *rigiduloides* diverged from those of *E*. *meliloti* strains, which nodulated other *Medicago* spp. [148,153], while the ability of certain strains of *Rhizobium leguminosarum* sv. *trifolii* to effectively nodulate *Trifolium ambiguum*, but not *Trifolium repens* (white clover) appears to be linked to a 111-bp insertion in their *nifH/fixA* intergenic *region* [315]. The factors that have resulted in these highly specific relationships are not known. Furthermore, it is not known if these highly specific relationships reflect adaptation of the symbioses and result in greater rates of N_2_ fixation or more efficient N_2_ fixation, as would be expected on theoretical grounds [322], and this warrants further study.

## 7. Conclusions

Overall, the data indicate that lateral gene transfer of specific symbiosis genes within rhizobial genera is an important mechanism allowing legumes to form symbioses with rhizobia adapted to particular soils. It also maintains specificity between legume species and rhizobia species with specific symbiosis genes. Strain-specific legume rhizobia symbioses can develop in particular habitats.

## Figures and Tables

**Table 1:** Legume-rhizobia symbioses in the legume sub-family Mimosoideae. All species have indeterminate nodules.

Mimosoideae Tribes and Genera	Rhizobia-Field
Ingeae	
*Acacia auriculiformis*	*Bradyrhizobium* [40,41,42]
*Acacia mangium*	*Bradyrhizobium* [40,41,43,44], *Ochrobactrum* [44], *Rhizobium* [44]
*Acacia mangium × A. auriculiformis*	*Bradyrhizobium* [43]
*Acacia mearnsii*	*Ensifer* [45,46]
*Acacia melanoxylon*	*Bradyrhizobium* [47]
*Acacia saligna*	*Bradyrhizobium* [42,48], *Ensifer* [49], *Rhizobium* [48]
*Acaciella angustissima*	*Ensifer* [50,51]
*Calliandra calothyrsis*	*Ensifer* [52], *Rhizobium* [52]
*Calliandra grandiflora*	*Ensifer* [53], *Mesorhizobium* [53], *Rhizobium* [53]
*Faidherbia albida*	*Bradyrhizobium* [54]
*Inga edulis*	*Bradyrhizobium* [55]
*Inga laurina*	*Bradyrhizobium* [56]
*Mariosousa acatlensis*	*Ensifer* [57]
*Senegalia laeta*	*Ensifer* [45]
*Senegalia macilenta*	*Ensifer* [57],
*Senegalia senegal*	*Ensifer* [45,46], *Rhizobium* [45,58], *Mesorhizobium* [58]
Mimoseae	
*Anadenanthera peregrina*	*Burkholderia* [59]
*Desmanthus illinoensis*	*Rhizobium* [60]
*Desmanthus paspalaceus*	*Mesorhizobium* [61], *Rhizobium* [61]
*Desmanthus virgatus*	*Rhizobium* [43]
*Leucaena leucocephala*	*Ensifer* [52,62,63], *Mesorhizobium* [52,62,63], *Rhizobium* [52,62,64]
*Microlobius foetidus*	*Bradyrhizobium* [59], *Rhizobium* [59]
*~50 Mimosa* spp.	*Burkholderia* [65,66,67,68,69,70,71,72,73,74,75,76,77]
*Mimosa affinis*	*Rhizobium* [72]
*Mimosa albida*, *M. biuncifera*, *M.borealis*, *M. dysocarpa*, *M. polyantha*, *M. tricephala*, *Mimosa* sp.	*Ensifer* [74], *Rhizobium* [74]
*Mimosa asperata*	*Cupriavidus* [78]
*Mimosa benthamii*, *M. goldmanii*, *M. monancistra*, *M. robusta*, *M. tequilana*	*Rhizobium* [74]
*Mimosa borealis*, *M. lacerata*, *M. luisana*, *M. similis*	*Ensifer* [74]
*Mimosa ceratonia*	*Rhizobium* [72]
*Mimosa cruenta*, *M. magentea*, *M. ramulosa*, *M. reptans*, *M. schleidenii*	*Cupriavidus* [79]
*Mimosa diplotricha*	*Burkholderia* [74], *Cupriavidus* [65,75,76], *Rhizobium* [65,72]
*Mimosa hamata*, *M. himalayana*	*Ensifer* [77]
*Mimosa invisa*	*Rhizobium* [70]
*Mimosa pigra*	*Burkholderia* [68], *Cupriavidus* [67,69]
*Mimosa polyantha*	*Rhizobium* [74]
*Mimosa pudica*	*Bradyrhizobium* [70], *Burkholderia* [74], *Cupriavidus* [65,69,72,75,76,77], *Rhizobium* [69,70]
*Mimosa skinneri*	*Burkholderia* [74], *Rhizobium* [74]
*Mimosa strigillosa*	*Ensifer* [78]
*Neptunia natans*	*Allorhizobium* [80], *Devosia* [81]
*Parapiptadenia pterosperma*	*Burkholderia* [59]
*Parapiptadenia rigida*	*Burkholderia* [59], *Cupriavidus* [82], *Rhizobium* [59]
*Piptadenia adiantoides*, *P. flava*	*Rhizobium* [59]
*Piptadenia gonoacantha*, *P. paniculata*	*Burkholderia* [59], *Rhizobium* [59]
*Piptadenia stipulacea*, *P. trisperma*, *P. vividiflora*	*Burkholderia* [59]
*Prosopis alba*	*Bradyrhizobium* [83], Ensifer [83,84], *Mesorhizobium* [83,85], *Rhizobium* [84]
*Prosopis chilensis*	*Ensifer* [46,86]
*Prosopis cineraria*	*Ensifer* [87]
*Prosopis farcta*	*Ensifer* [88], *Mesorhizobium* [88]
*Prosopis juliflora*	*Ensifer* [35], *Rhizobium* [35]
*Pseudopiptadenia contorta*	*Burkholderia* [59]
*Stryphnodendron* sp.	*Bradyrhizobium* [59]
*Vachellia abyssinica*	*Mesorhizobium* [89], *Ensifer* [90]
*Vachellia cochliacantha*, *V. farnesiana*, *V. pennatula*	*Ensifer* [57]
*Vachellia gummifera*	*Ensifer* [49]
*Vachellia horrida*	*Ensifer* [45,49]
*Vachellia jacquemontii*	*Ensifer* [87,91]
*Vachellia macracantha*	*Ensifer* [92], *Rhizobium* [92]
*Vachellia nubica*	*Bradyrhizobium* [54]
*Vachellia seyal*	*Rhizobium* [45], *Ensifer* [90]
*Vachellia tortilis*	*Ensifer* [45,49,90,93], *Mesorhizobium* [45,54,89,93], *Rhizobium* [93]
*Vachellia xanthophloea*	*Mesorhizobium* [54]
*Xylia xylocarpa*	*Bradyrhizobium* [40,43]

**Table 2:** Legume-rhizobia symbioses in the inverted repeat lacking clade (IRLC) of the legume sub-family Papilionoideae. All species in the IRLC have indeterminate nodules.

Papilionoidieae Tribes and Genera	Rhizobia-Field
Cicereae	
*Cicer arietinum*	*Mesorhizobium* [94,95,96,97,98,99,100,101]
*Cicer canariense*	*Mesorhizobium* [102]
Fabeae	
*Lathyrus aphaca*, *L. nissolia*, *L. pratensis*	*Rhizobium* [103]
*Lathyrus japonicus*	*Rhizobium* [104]
*Lathyrus odoratus*	*Rhizobium* [105]
*Lens culinaris*	*Rhizobium* [101,106,107]
*Pisum sativum*	*Rhizobium* [101,103,107,108]
*Vicia amoena*, *V. bungei*, *V. villosa*	*Rhizobium* [109]
*Vicia cracca*	*Rhizobium* [103,109,110]
*Vicia hirsuta*	*Rhizobium* [103,105,110]
*Vicia faba*	*Rhizobium* [101,103,108,109,111,112,113,114]
*Vicia multicaulis*, *V. sylvatica*, *V. tetrasperma*	*Rhizobium* [110]
*Vicia sativa*	*Rhizobium* [103,109,115,116,117,118]
*Vicia sepium*	*Rhizobium* [109,110]
Galega	
*Galega officinalis*	*Neorhizobium* [119,120]
*Galega orientalis*	*Neorhizobium* [119]
Galegeae	
*Astragalus adsurgense*	*Ensifer* [121], *Mesorhizobium* [121], *Rhizobium* [122]
*Astragalus aksuensis*, *A. betetovii*	*Rhizobium* [105]
*Astragalus complanatus*	*Ensifer* [121], *Mesorhizobium* [121], *Rhizobium* [122]
*Astragalus chrysopterus*	*Rhizobium* [122]
*Astragalus discolor*, *A. efoliolatus*, *A. kifonsanicus*	*Mesorhizobium* [121]
*Astragalus melilotoides*	*Ensifer* [121], *Mesorhizobium* [121]
*Astragalus membranaceus*	*Mesorhizobium* [121,123,124]
*Astragalus mongholicus*	*Mesorhizobium* [124]
*Astragalus polycladus*	*Rhizobium* [121]
*Astragalus scaberrimus*	*Mesorhizobium* [121], *Rhizobium* [122]
*Biserrula pelecinus*	*Mesorhizobium* [125,126]
*Carmichaelia australis*, *C. monroi*,	*Mesorhizobium* [127]
*Clianthus puniceus*	*Mesorhizobium* [127]
*Colutea arborescens*	*Ensifer* [128], *Mesorhizobium* [128,129], *Rhizobium* [128]
*Glycyrrhiza eurycarpa*	*Ensifer* [130]
*Glycyrrhiza glabra*	*Mesorhizobium* [130,131], *Rhizobium* [130]
*Glycyrrhiza inflata*	*Ensifer* [130]
*Glycyrrhiza multiflora*	*Mesorhizobium* [132]
*Glycyrrhiza pallidiflora*	*Mesorhizobium* [133]
*Glycyrrhiza uralensis*	*Mesorhizobium* [130,132], *Rhizobium* [130]
*Glycyrrhiza* sp.	*Mesorhizobium* [130]
*Gueldenstaedtia multiflora*	*Mesorhizobium* [132], *Rhizobium* [132,134]
*Lessertia annulans*, *L. capitata*, *L. diffusa*, *L. excisa*, *L. frutescens*, *L. herbacea*, *L. microphylla*, *L. pauciflora*	*Mesorhizobium* [135]
*Lessertia* sp.	*Ensifer* [136]
*Montigena novae-zelandiae*	*Mesorhizobium* [137]
*Oxytropis glabra*	*Ensifer* [109], *Mesorhizobium* [138], *Rhizobium* [105,109]
*Oxytropis kansuenses*, *O. myriophylla*, *O. psammocharis*	*Rhizobium* [109]
*Oxytropis meinshausenii*	*Rhizobium* [105]
*Oxytropis ochrocephala*	*Mesorhizobium* [109], *Rhizobium* [109]
*Oxytropis* sp.	*Phyllobacterium* [109]
*Sphaerophysa salsula*	*Ensifer* [139], *Mesorhizobium* [139], *Rhizobium* [139,140]
*Swainsona leeana*, *S. pterostylis*	*Ensifer* [141]
*Swainsona galegifolia*	*Mesorhizobium* [137]
Hedysareae	
*Alhagi sparsifolia*	*Mesorhizobium* [142]
*Alhagi toum*	*Rhizobium* [105]
*Caragana bicolor*, *C. erinacea*	*Mesorhizobium*, *Rhizobium* [143]
*Caragana franchetiana*	*Mesorhizobium*, [143]
*Caragana intermedia*	*Bradyrhizobium* [143], *Mesorhizobium* [132,143], *Rhizobium* [143]
*Caragana jubata*	*Rhizobium* [105]
*Caragana microphylla*	*Mesorhizobium* [144]
*Halimodendron halodendron*	*Rhizobium* [105]
*Hedysarum coronarium*	*Rhizobium* [120,145]
*Hedysarum polybotrys*	*Rhizobium* [122], *Mesorhizobium* [124]
*Hedysarum scoparium*	*Rhizobium* [122]
*Hedysarum spinosissimum*	*Ensifer* [118]
*Onobrychis viciifolia*	*Phyllobacterium* [146]
Trifolieae	
*Medicago archiducis-nicolai*	*Rhizobium* [109]
*Medicago intertexta*	*Ensifer* [147]
*Medicago laciniata*	*Ensifer* [147,148,149,150], *Neorhizobium* [147]
*Medicago lupulina*	*Ensifer* [109,151]
*Medicago orbicularis*	*Ensifer* [152]
*Medicago polymorpha*	*Ensifer* [147], *Neorhizobium* [147]
*Medicago rigiduloides*	*Ensifer* [153]
*Medicago ruthenica*	*Rhizobium* [154]
*Medicago sativa*	*Ensifer* [109,150,155,156,157,158], *Neorhizobium* [147], *Rhizobium* [156]
*Medicago scutellata*	*Ensifer* [150]
*Medicago truncatula*	*Ensifer* [149,150,152]
*Melilotus alba*	*Ensifer* [156], *Rhizobium* [156]
*Melilotus indicus*, *M. messanensis*, *M. siculus*	*Ensifer* [147]
*Melilotus officinalis*	*Ensifer* [109,151]
*Trigonella maritima*	*Ensifer* [118,147]
Trifolium	
*Trifolium fragiferum*	*Bradyrhizobium* [70], *Mesorhizobium* [70], *Rhizobium* [70]
*Trifolium pratense*	*Phyllobacterium* [159],
*Trifolium repens*	*Bradyrhizobium* [70], *Ensifer* [70], *Rhizobium* [70,160]

**Table 3:** Legume-rhizobia symbioses of species in the sub-family Papilionoideae with indeterminate nodules excluding the inverted repeat lacking clade.

Papilionoideae Tribes (Genera)	Rhizobia-Field
Abreae	
*Abrus precatorius*	*Ensifer* [161]
Amorpheae	
*Amorpha fruticosa*	*Bradyrhizobium* [162], *Mesorhizobium* [132,151,162]
*Dalea purpurea*	*Mesorhizobium* [163], *Rhizobium* [163]
Crotalarieae	
*Aspalathus callosa*	*Burkholderia* [136]
*Aspalathus ciliaris*, *A. unifllora*	*Mesorhizobium* [136]
*Aspalathus linearis*	*Bradyrhizobium* [36], *Burkholderia* [36], *Mesorhizobium* [36], *Rhizobium* [36]
*Crotalaria comosa*, *C. hyssopifolia*, *C. lathyroides*	*Bradyrhizobium* [164]
*Crotalaria pallida*	*Bradyrhizobium* [70], *Burkholderia* [70], *Rhizobium* [70]
*Crotalaria perrotteti*, *C. podocarpa*	*Methylobacterium* [164]
*Crotalaria* sp.	*Burkholderia* [136]
*Lebeckia ambigua*	*Burkholderia* [165]
*Listia angolensis*	*Microvirga* [166]
*Listia bainesii*, *L. solitudinis*, *L. listii*	*Methylobacterium* [16]
*Lotononis laxa*, *L. sparsifolia*	*Ensifer* [17]
*Lotononis* sp.	*Bradyrhizobium*, *Mesorhizobium* [17]
*Rafnia* sp.	*Burkholderia* [136]
Genisteae	
*Adenocarpus hispanicus*	*Phyllobacterium* [129]
*Argyrolobium uniflorum*	*Ensifer* [150,157]
*Argyrolobium* sp.	*Mesorhizobium* [136]
*Cytisus aeolicus*	*Bradyrhizobium* [167]
*Cytisus balansae*, *C. multiflorus*, *C. striatus*	*Bradyrhizobium* [168]
*Cytisus laburnum*, *C. purgans*	*Bradyrhizobium* [129]
*Cytisus proliferus*	*Bradyrhizobium* [169,170,171,172]
*Cytisus scoparius*	*Bradyrhizobium* [173,174]
*Cytisus villosus*	*Bradyrhizobium* [175]
*Genista hystrix*	*Bradyrhizobium* [168]
*Genista stenopetula*	*Bradyrhizobium* [170]
*Genista versicolor*	*Bradyrhizobium* [176]
*Lupinus albescens*	*Bradyrhizobium* [177,178]
*Lupinus albus*	*Bradyrhizobium* [172,179,180]
*Lupinus angustifolius*	*Bradyrhizobium* [172,180]
*Lupinus honoratus*	*Ochrobactrum* [181]
*Lupinus luteus*	*Bradyrhizobium* [172,180]
*Lupinus mariae-josephae*	*Bradyrhizobium* [182,183]
*Lupinus montanus*	*Bradyrhizobium* [170]
*Lupinus micranthus*	*Bradyrhizobium* [184]
*Lupinus polyphyllus*	*Bradyrhizobium* [170,185]
*Lupinus texensis*	*Microvirga* [166]
*Lupinus* sp.	*Bradyrhizobium* [172]
*Retama monosperma*	*Bradyrhizobium* [186]
*Retama raetam*	*Bradyrhizobium* [187]
*Retama sphaerocarpa*	*Bradyrhizobium* [168,186,187,188], *Phyllobacterium* [129]
*Spartium junceum*	*Bradyrhizobium* [129,167,189], *Phyllobacterium* [129]
*Ulex europaeus*	*Bradyrhizobium* [190]
Hypocalypteae	
*Hypocalyptus coluteoides*, *H. oxalidifolius*, *H. sophoroides*	*Burkholderia* [191]
Indigofereae	
*Indigofera angustifolia*	*Burkholderia* [136]
*Indigofera astragalina*, *I. hirsuta*, *I. senegalensis*, *I. tinctoria*	*Bradyrhizobium* [192]
*Indigofera filifolia*	*Burkholderia* [193]
Loteae	
*Coronilla varia*	*Mesorhizobium* [194], *Rhizobium* [132,134,194]
*Ornithopus compressus*, *O. sativus*	*Bradyrhizobium* [195]
Millettieae	
*Milletia leucantha*	*Bradyrhizobium* [40]
*Millettia pinnata*	*Rhizobium* [196]
*Tephrosia capensis*	*Bradyrhizobium* [136]
*Tephrosia falciformis*	*Bradyrhizobium* [87], *Ensifer* [87]
*Tephrosia purpurea*	*Bradyrhizobium* [192], *Ensifer* [87], *Rhizobium* [87]
*Tephrosia villosa*	*Bradyrhizobium* [87,192], *Ensifer* [87]
*Tephrosia wallichii*	*Ensifer* [86]
Podalyrieae	
*Cyclopia buxifolia*, *C. genistoides*, *C. glabra*, *C. intemedia*, *C. longifolia*, *C. maculata*, *C. meyeriana*, *C. pubescens*, *C. sessiflora*, *C. subternata*	*Burkholderia* [191]
*Podalyria burchelli*, *P. sericea*	*Burkholderia* [136]
*Podalyria calyptrata*	*Burkholderia* [136,191,193,197]
*Podalyria pinnata*	*Burkholderia* [193]
*Virgilia divaricata*	*Rhizobium* [136]
*Virgilia oroboides*	*Burkholderia* [136,191]
Robineae	
*Gliricidia sepium*	*Ensifer* [52], *Rhizobium* [52]
*Robinia pseudocacia*	*Mesorhizobium* [198,199], *Rhizobium* [105,198]
Sesbanieae	
*Sesbania aculeata*, *S. grandiflora*, *S. pachycarpa*, *Sesbania* sp.	*Ensifer* [45]
*Sesbania cannabina*	*Ensifer* [45,200,201,202], *Neorhizobium* [202], *Rhizobium* [200,202]
*Sesbania exasperata*	*Rhizobium* [30]
*Sesbania herbacea*	*Rhizobium* [203]
*Sesbania punicea*	*Azorhizobium* [136,204], *Mesorhizobium* [30], *Rhizobium* [204]
*Sesbania rostrata*	*Azorhizobium* [205,206], *Bradyrhizobium* [43], *Ensifer* [45,161], *Rhizobium* [43]
*Sesbania sericea*	*Mesorhizobium* [30], *Rhizobium* [30]
*Sesbania sesban*	*Ensifer* [45,52,90], *Mesorhizobium* [52,54,89], *Rhizobium* [52,54]
*Sesbania virgata*	*Azorhizobium* [206], *Rhizobium* [204]
Sophoreae	
*Sophora alopecuroides*	*Ensifer* [207], *Mesorhizobium* [207], *Phyllobacterium* [207], *Rhizobium* [105,207]
*Sophora flavescens*	*Bradyrhizobium* [208], *Ensifer* [208], *Mesorhizobium* [208], *Phyllobacterium* [209], *Rhizobium* [208]
*Sophora longicarinata*, *S. microphylla*, *S. prostrata*, *S. tetraptera*	*Mesorhizobium* [210]
*Sophora viciifolia*	*Mesorhizobium* [132]
Thermopsideae	
*Ammopiptanthus nanus*, *A. mongolicus*	*Ensifer* [211], *Neorhizobium* [211], *Pararhizobium* [211], *Rhizobium* [211]
*Anagyris latifolia*	*Mesorhizobium* [212]
*Thermopsis lupinoides*	*Mesorhizobium* [213]

**Table 4:** Legume-rhizobia symbioses of species in the sub-family Papilionoideae with determinate nodules.

Papilionoideae Tribes and Genera	Rhizobia-Field
Dalbergieae	
*Adesmia bicolor*	*Rhizobium* [214]
*Aeschynomene afraspera*, *A. ciliata*, *A. elaphroxylon*, *A. scabra*, *A. sensitiva*, *A. shimperi*	*Bradyrhizobium* [34]
*Aeschynomene americana*	*Bradyrhizobium* [34,215]
*Aeschynomene indica*	*Bradyrhizobium* [34,216]
*Aeschynomene rudis*	*Bradyrhizobium* [34,217]
*Arachis duranensis*	*Bradyrhizobium* [218]
*Arachis hypogaea*	*Bradyrhizobium* [43,219,220,221,222,223,224,225,226,227,228,229], *Rhizobium* [221,222]
*Centrolobium paraense*	*Bradyrhizobium* [230,231]
*Dalbergia baroni*, *D. louveli*, *D. madagascariensis*, *D. maritima*, *D. monticola*, *D. purpurascens*, *Dalbergia* sp.	*Bradyrhizobium* [232]
*Pterocarpus officinalis*	*Bradyrhizobium* [233]
*Pterocarpus indicus*	*Bradyrhizobium* [40,43]
*Zornia glochidiata*	*Bradyrhizobium* [234]
Desmodieae	
*Desmodium caudatum*, *D. fallax*, *D. triflorum*	*Bradyrhizobium* [235]
*Desmodium elegans*	*Bradyrhizobium* [235,236], *Pararhizobium* [236]
*Desmodium gangeticum*	*Bradyrhizobium* [235,237]
*Desmodium heterocarpan*	*Bradyrhizobium* [235,237]
*Desmodium microphyllum*	*Bradyrhizobium* [235], *Mesorhizobium* [235], *Rhizobium* [235]
*Desmodium oldhami*	*Rhizobium* [236]
*Desmodium racemosum*	*Bradyrhizobium* [235], *Ensifer* [235], *Rhizobium* [235]
*Desmodium sequax*	*Bradyrhizobium* [235], *Ensifer* [235], *Mesorhizobium* [236], *Pararhizobium* [236], *Rhizobium* [235,236]
*Desmodium sinuatum*	*Rhizobium* [238]
*Kummerowia stipulacea*	*Bradyrhizobium* [151,239], *Rhizobium* [239]
*Kummerowia striata*	*Bradyrhizobium* [239], *Ensifer* [239], *Rhizobium* [239]
*Lespedeza bicolor*	*Bradyrhizobium* [240], *Ensifer* [240], *Mesorhizobium* [151] *Rhizobium* [240]
*Lespedeza capitata*, *L. cuneata*, *L. juncea*, *L. procumbens*, *L. stipulacea*, *L. striata*	*Bradyrhizobium* [240]
*Lespedeza cystobotrya*	*Ensifer* [240], *Rhizobium* [122]
*Lespedeza daurica*	*Bradyrhizobium* [240], *Ensifer* [240], *Mesorhizobium* [240]
*Lespedeza davidii*	*Rhizobium* [122]
*Lespedeza inschanica*, *L. tomentosa*	*Ensifer* [240]
Phaseoleae	
*Amphicarpaea bracteata*, *A. edgeworthii*	*Bradyrhizobium* [241]
*Amphicarpaea trisperma*	*Rhizobium* [132]
*Bolusafra bituminosa*	*Burkholderia* [136]
*Cajanus cajan*	*Bradyrhizobium* [242]
*Canavalia rosea*	*Ensifer* [243]
*Centrosema pascuorum*	*Bradyrhizobium* [43]
*Centrosema pubescens*	*Bradyrhizobium* [42]
*Dipogon lignosus*	*Burkholderia* [15,193]
*Glycine max*	*Bradyrhizobium* [43,151,244,245,246,247,248,249,250,251], *Ensifer* [245,246,247,252,253], *Rhizobium* [254]
*Glycine soja*	*Bradyrhizobium* [151,255], *Ensifer* [255], *Rhizobium* [256]
*Lablab purpureus*	*Bradyrhizobium* [224,226,227]
*Neonotonia wightii*	*Bradyrhizobium* [237]
*Pachyrhizus erosus*	*Bradyrhizobium* [257,258,259], *Rhizobium* [257]
*Pachyrhizus ferrugineus*, *P. tuberosus*	*Bradyrhizobium* [258]
*Phaseolus lunatus*	*Bradyrhizobium* [260,261,262], *Rhizobium* [262]
*Phaseolus vulgaris*	*Bradyrhizobium* [260,263], *Burkholderia* [264,265], *Ensifer* [263,266,267,268], *Pararhizobium* [263], *Rhizobium* [64,101,151,227,263,266,267,268,269,270,271,272,273,274,275]
*Pueraria phaseoloides*	*Bradyrhizobium* [276]
*Rhynchosia aurea*	*Ensifer* [87]
*Rhynchosia ferulifolia*	*Burkholderia* [277,278]
*Rhynchosia minima*	*Bradyrhizobium* [192,277]
*Rhynchosia totta*	*Bradyrhizobium* [277]
*Vigna angularis*	*Bradyrhizobium* [279], *Ensifer* [279], *Rhizobium* [279]
*Vigna radiata*	*Bradyrhizobium* [280,281], *Ensifer* [280], *Rhizobium* [280]
*Vigna sinensis*	*Bradyrhizobium* [43]
*Vigna subterranea*	*Bradyrhizobium* [227,282], *Burkholderia* [282], *Rhizobium* [282]
*Vigna unguiculata*	*Bradyrhizobium* [223,227,280,283,284,285], *Burkholderia* [283], *Microvirga* [286], *Rhizobium* [280,283]
Psoraleae	
*Otholobium bracteolatum*, *O. hirtum*, *O. virgatum*, *O. zeyhari*, *Otholobium* sp.	*Mesorhizobium* [136]
*Psoralea asarina*	*Burkholderia* [286]
*Psoralea corylifolia*	*Ensifer* [201]
*Psoralea pinnata*	*Bradyrhizobium* [193], *Burkholderia* [287], *Mesorhizobium* [136,193,287]
Loteae	
*Lotus arabicus*, *L. arinagensis*	*Ensifer* [157]
*Lotus bertheloti*, *L. callis-viridis*, *L. campylocladus*, *L. pyranthus*	*Mesorhizobium* [288]
*Lotus corniculatus*	*Mesorhizobium* [39,288,289,290,291]
*Lotus creticus*	*Ensifer* [118,157], *Mesorhizobium* [118,157], *Rhizobium* [118,157]
*Lotus frondosus*	*Mesorhizobium* [138], *Rhizobium* [105]
*Lotus halophyllus*	*Ensifer* [118]
*Lotus kunkeli*, *L. lancerottensis*, *L. maculatus*	*Ensifer* [292]
*Lotus sessilifolius*	*Ensifer* [292], *Mesorhizobium* [288]
*Lotus tenuis*	*Mesorhizobium* [291,293,294], *Rhizobium* [105,293]
*Lotus uliginosus*	*Bradyrhizobium* [295]
